# Food selection criteria for disaster response planning in urban societies

**DOI:** 10.1186/s12937-015-0033-0

**Published:** 2015-05-12

**Authors:** Michelle Wien, Joan Sabaté

**Affiliations:** 1 Human Nutrition and Food Science Department, College of Agriculture, California State Polytechnic University, Pomona, 3801 West Temple Avenue, Pomona, CA 91768 USA; 2 Loma Linda University, School of Public Health, Loma Linda, CA 92350 USA

**Keywords:** Disaster management, Nutrient density, Naturally nutrient rich score, Cultural acceptance, Plant-based foods

## Abstract

**Background:**

Nutrition professionals that have menu planning and disaster management responsibilities should consider factors that have transcended from ancient to current times, in addition to recognizing societal trends that have led to our current increased vulnerability in the event of a disaster. Hence, we proceeded to develop a set of “Disaster Response Diets” (DRDs) for use in urban societies inclusive of the aforementioned considerations.

**Methods:**

A three-phase multidimensional approach was used to identify food groups suitable for creating a set of DRDs. Phase One consisted of calculating the percent daily nutrient intake and Drewnowski’s naturally nutrient rich (NNR) score for an individual or mean composite for one serving of food from 11 specific food groups. In Phase Two, in addition to nutrient density, the 11 food groups were evaluated and scored based on the following DRD planning criteria: storage and handling properties, preparation ease and, cultural acceptance/individual tolerance. During Phase Three, three DRDs were developed based upon the data retrieved from Phases one and two.

**Results:**

In Phase One, the NNR scores ranged from 2.1 for fresh fruits to 28.1 for dry cereals, a higher score indicating a higher nutrient density. During Phase Two, a maximum score of 12 was possible based on appropriateness for a disaster situation. Five plant-based food groups (dry cereals, nuts, dried fruits, grains and legumes) achieved a score ranging between 7 and 12, whereas the five fresh food groups were deemed ineligible due to sanitation and perishability concerns. During Phase Three, three DRDs (milk-inclusive, milk-free and Grab-and-Go) were developed as benchmarks for disaster response planning.

**Conclusions:**

Plant-based DRDs are universally acceptable and tolerated across cultures and religions. Therefore, we suggest nutrition professionals consider using a plant-based approach for creating DRDs for public health institutions and organizations.

## Background

Natural and man-made disasters are a part of the human experience. However, the advent of instant global communication now gives more attention to these events than in previous generations. Nutrition professionals that have menu planning and disaster management responsibilities should consider factors that have transcended from ancient to current times, in addition to recognizing societal trends that have led to our current increased vulnerability in the event of a disaster.

For centuries individuals residing in rural agricultural communities lived in isolation and had limited food availability. Individuals in these communities or geographic regions were homogeneous in culture and race (e.g., consistent gene pool) and were naturally forced to plan ahead for periods of potential famine caused by natural disasters (e.g., drought, floods, and fires) or erratic weather patterns (e.g., heavy rain or snow). A combination of grains, nuts, seeds, dried fruits and/or dried meats met their food selection criteria as these foods were not prone to spoilage. Additionally, the stored foods were culturally acceptable, well tolerated, and did not conflict with social taboos or religious proscriptions [1]. More recently, vast urbanization has created a global melting pot which adds an additional layer of complexity for selecting religious, cultural and ethnically acceptable foods in disaster planning. Dietary norms are present in most religions among many segments of populations within developed and developing countries. Furthermore, some these precepts clearly state what, how and when to eat or avoid certain foods [2].

The migratory pattern from the rural to urban settings over the past few decades has increased the vulnerability among most urban societies when planning for disaster preparedness (Table 1). In 1990 less than 40 % of the global population resided in an urban setting as compared to 53 % in 2012 [3]. Other contributing lifestyle changes in urban settings are the increased consumption of fresh produce, convenience foods and, eating away from home. According to a 2009 survey, there is an increased trend in eating fresh, green and/or local food [4], which further perpetuates vulnerability because these foods require refrigeration. As the urban population continues to increase households have less non-perishable food in storage and fewer storage areas [5]. Food transportation and distribution systems have also greatly improved over the past century. This has favorably influenced food patterns worldwide by adding fresh fruits and vegetables to dietary patterns as well as complete frozen meals [6]. The combination of expanding global markets and efficient distribution systems has added variety, freshness and convenience to household inventories, yet has simultaneously contributed to a higher proportion of perishable foods that are on hand in the event of a disaster. Additionally, the reliance on refrigerators and freezers has made individuals and societies more vulnerable to disasters that suddenly disrupt food distribution and electrical grids [7]. Moreover, many individuals eat one or more daily meals away from home at restaurants, schools, work cafeterias, etc. [8] thus reducing the home inventory of non-perishable food items stored in household pantries. As frozen foods, complete frozen meals [6] and fresh foods from farmer’s markets become increasingly popular, newly constructed homes feature more space in kitchens for larger refrigerators and freezers and less pantry storage capacity. Institutional cafeterias (e.g., hospitals, schools, prisons) and restaurants also store the majority of food in their freezers and refrigerators [9] which further diminishes the supply of non-perishable food staples for use during a disaster.

**Table 1:** Trends in urban societies that increase vulnerability in the event of a disaster

Trend	Vulnerability outcome
Eating fresh, green and/or local [4]; Increased use of fresh fruit and vegetables [6]	High proportion of home food inventory is stored in refrigerator(s)
Frequently eating away from home [8]	Lower inventory of food at home
Convenience foods [5]	High proportion of home food inventory is stored in freezer(s)
Apartment/urban living conditions [3]	Limited food storage space

The United States (US) Federal Emergency Management Agency (FEMA) has provided guidelines for the recommended levels of food and water in an emergency [10]. Government agencies traditionally publish recommendations for disaster preparedness primarily based on energy and key nutrient content; we, however, propose a three-phase multidimensional approach to identify which food groups are suitable for developing “Disaster Response Diets” (DRDs) for the home, institutions, government agencies and non-governmental organizations (NGOs).

Nutrient density is commonly defined as the ratio of the nutrient content to the total energy content of a given food item. Beyond nutrient density, it is important to consider a food’s storage and handling properties, preparation ease and cultural acceptability/tolerance among the population being served. FEMA suggests the inclusion of foods that have a shelf-life ranging from six months to indefinitely [10]. Therefore, foods that are shelf stable, do not require refrigeration or freezing and have minimal sanitation concerns in their natural or reconstituted state are most preferable for disaster planning.

This paper will describe a three-phase multidimensional approach to identify the specific food groups based upon the aforementioned criteria for developing a set of DRDs for nutrition professionals to use in urban societies. It is beyond the scope of this paper to address the nutritional concerns of severely malnourished children and adults in developing nations that are fleeing adverse conditions or to describe how to set up temporary kitchens, determine staffing needs and budgeting, which are also important considerations for disaster response management.

## Methods

### Nutrient density (phase one)

The percent daily values (%DV) listed on US food labels are used to guide consumers in menu planning. These %DV are based on the Dietary Reference Intakes (DRIs) for an intake of 2000 calories per day [11]. The number of foods selected for a food group ranged between one and five and the individual or mean composite nutrient content of the food groups was computed to determine the %DV. More specifically, the dry cereal group included Corn Flakes®, Cheerios®, Shredded Wheat® and granola; the nuts group featured almonds, walnuts, cashews, pecans and pistachios; the dried fruit group consisted of apricots, figs, raisins, apples and dates; the grains group included white rice, saltine crackers, cream of wheat and enriched pasta; the legumes group included peas, lentils, soybeans, kidney beans and chickpeas; the dried meat/fish group included beef and cod; the fresh vegetables group consisted of carrots, tomato, spinach, celery and bell pepper; the fresh fruit group included apple, orange, banana, seedless grapes and cantaloupe; and, the fresh meat/fish group featured ground beef, pork roast, cod, chicken and salmon.

In order to determine nutrient density with the various chosen food groups, Drewnowski’s naturally nutrient rich (NNR) score was used to evaluate the nutrient density using the formula Σ%DV_2000kcal_/14. The NNR score numerically evaluates the nutrient-to calorie ratio based on the following 14 key nutrients: protein, vitamins A, D, E, C, B-1, B-2, and B-12, folate, calcium, iron, zinc, monounsaturated fatty acids, and potassium [12]. The percent daily nutrient intake and the NNR are the components of a nutrient rich diet as defined by Drewnowski [12]. The scores for an individual or mean composite for one serving of food were computed for the 11 food groups [dry cereals, nuts, dried fruits, grains, legumes, dried meat/fish, dry nonfat (NF) milk, fresh vegetables, fresh fruits, fresh low fat (LF) milk, eggs and fresh meat/fish] in order to determine their value. The %DV for the nutrient analysis of foods within these food groups was obtained from the US Department of Agriculture, Agricultural Research Service’s National Nutrient Database for Standard Reference, Release 26 [13].

### Scoring system (phase two)

The 11 food groups were scored according to the following four criteria: 1) nutrient density; 2) storage and handling properties; 3) preparation ease; and, 4) cultural acceptance/individual tolerance. Food groups received a score ranging from zero to three for each of the equally weighted four food selection criteria; hence, a total score of 12 points was possible. For nutrient density, a score of zero was given to foods with a NNR score of 0–3.50, one point for a NNR score of 3.51–7.00, two points for a NNR score of 7.01–10.50, and three points for a NNR score greater than 10.50. For storage and handling properties, foods that require little storage space (e.g., dried, compact and/or lightweight) received one point whereas foods with high moisture content were given zero points. A shelf life of 12 or more months was the operational cut-off established to receive one point in contrast to foods with a short shelf life, which received a score of zero. “Fresh” fruits, vegetables, meat and milk require refrigeration and have a higher degree of sanitation and perishability concerns. Therefore, “fresh” foods were deemed ineligible for inclusion in developing the set of DRDs. For preparation ease, if no utensils were required for food preparation or consumption, one point was provided. Foods that require cooking received a zero in contrast to those that are ready for immediate consumption, which received one point. If a food was dependent upon water for reconstitution it received zero points. If a food was culturally accepted and did not pose concerns related to allergies or gastrointestinal intolerance, a score of three was given. A score of two was the highest score achievable if there were concerns for allergies or gastrointestinal intolerance and foods that are unacceptable to some cultures received a score of zero. In light of the magnitude of social taboos and religious prohibition of meat and fish among some vegetarian societies (e.g., Jainism, Hinduism and Seventh-day Adventist), the dried meat/fish group was also deemed ineligible for inclusion in developing the set of DRDs.

### Disaster response diets (phase three)

The operational definition for developing the DRDs was that they had to provide approximately 40–50 % of daily energy requirements (see rationale below) and achieve a minimum of 40 % of the %DV for at least nine of the 14 nutrients from the NNR score. This also included a subset of seven nutrients that comprise the Nutrient Rich Food Index [14]. Additionally, the DRDs must be comprised of foods with efficient storage and handling properties, be Grab-and-Go (no utensils, cooking or reconstitution required for the days immediately following a disaster while longer term foodservice systems are being established for cooking specific cereals, grains and legumes) and be culturally acceptable and tolerated by most individuals.

The rationale for the aforementioned operational definition for energy requirements is based on the evidence that a daily food ration that meets approximately 40 to 50 % of energy needs is sufficient to sustain life [15]. According to Elia [15] a healthy lean adult can survive 60–70 days without food when sufficient hydration is available; an obese adult can also live 200–300 days without food but must have an adequate water supply. Thus, an optimal combination of foods would include nutrient dense foods that can achieve this lower level of energy in the context of exceeding 50 % of the recommended intake levels for key nutrients. According to the National Health and Nutrition Examination Survey data between 1999–2002 in the US, approximately 65 % of the adult population is overweight or obese, and 31 % of children are either overweight or obese [16]. Based on the assumption that a daily food ration that meets approximately 50 % of energy needs is sufficient for the overweight/obese population at-large, the calorie needs per adult is closer to 1350 kcal daily [(0.35 × 2000 kcal for normal weight) + (0.65 × 1000 kcal for overweight/obese weight)] and 1690 kcal daily per child [(0.69 × 2000 kcal for normal weight) + (0.31 × 1000 for overweight/obese)].

## Results

### Nutrient density (phase one)

Table 2 shows the percent daily nutrient intake and NNR score for an individual or mean composite for one serving of food from the 11 distinct food groups.

**Table 2:** Percent daily nutrient intake and naturally nutrient rich (NNR) score for an individual or mean composite for 1 serving of food from 11 food groups

Nutrient	Dry cereal^a^	Nuts^b^	Dried fruit^c^	Grains^d^	Legumes^e^	Dried meat/fish^f^	Dry milk	Fresh vegetables^g^	Fresh fruits^h^	Fresh milk	Eggs	Fresh meat/fish^i^
Protein	3.1	7.3	1.4	3.2	12.2	16.7	12.4	1.7	1.62	12.6	9.7	34.5
Vitamin A	6.8	0.6	5.0	1.5	4.7	0.3	10.9	102.8	23.8	9.6	5.2	0.7
Vitamin D	200	0.0	0.0	60.0	0.0	5.5	25.0	0.0	0.0	29.0	7.0	2.6
Vitamin E	1.3	11.9	2.3	0.4	2.1	2.6	0.0	4.3	1.0	0.1	4.7	1.7
Vitamin C	3.4	0.6	0.6	1.2	18.5	0.5	1.7	43.1	39.9	0.0	0.0	0.3
Vitamin B-1	23.3	11.7	1.7	16.7	16.4	4.0	7.9	4.6	5.4	4.1	2.8	15.9
Vitamin B-2	21.8	6.6	2.6	11.5	6.4	3.3	30.8	4.0	4.6	34.7	19.8	9.8
Folate	28.7	3.4	0.8	13.6	30.2	4.1	3.0	8.0	5.9	3.0	5.5	1.2
Vitamin B-12	65.4	0.0	0.0	16.7	0.0	54.2	38.3	0.0	0.0	47.9	23.3	58.9
Calcium	2.1	2.5	2.0	3.1	2.7	1.6	21.8	2.2	1.6	23.5	1.9	1.2
Iron	19.8	6.2	3.5	17.6	12.9	4.7	0.4	13.2	1.6	0.4	3.3	5.8
Zinc	13.6	9.6	1.2	2.0	11.4	9.1	9.2	2.0	1.3	9.3	4.8	16.3
MUFA	1.3	36.4	0.1	0.6	2.1	5.8	0.2	0.4	0.1	3.4	10.2	13.2
Potassium	2.1	5.2	7.8	0.9	9.4	6.7	11.2	8.6	8.6	10.5	1.8	8.2
NNR Score	28.1	7.3	6.8	10.6	9.2	8.5	12.4	13.9	2.1	13.4	7.1	12.2

NNR score = Σ%DailyValue_2000kcal_/14; Monounsaturated fatty acid (MUFA)

^a^Dry Cereal: Cornflakes®, Cheerios® (3/4 cup); Shredded Wheat® (1/2 cup); granola (1/4 cup)

^b^Nuts: almonds, walnuts, cashews, pecans, pistachios (1 oz each)

^c^Dried Fruit: apricots, figs, raisins, apples, dates (1/4 cup each)

^d^Grains: cooked white rice (1/3 cup); 6 saltine crackers; cream of wheat, enriched pasta (1/2 cup cooked)

^e^Legumes: raw peas, raw soybeans (1 cup); canned kidney beans, cooked chick peas, cooked lentils (1/2 cup)

^f^Dried Meat/Fish: beef, cod (1 oz)

^g^Fresh vegetables: carrots, tomato, spinach, celery, bell pepper (1 cup)

^h^Fresh fruit: apple, orange, banana (1 piece); seedless grapes, cantaloupe (1 cup)

^i^Fresh Meat/Fish: ground beef, pork roast, cod, chicken, salmon (3 oz)

### Scoring system (phase two)

The results of the food group scoring are shown in Table 3. Grains received 9 points while legumes received 7 because they are both moderately compact in volume, have a long shelf life, require no refrigeration and are not prone to spoilage until after reconstitution. However, they do require a heat source for cooking, cooking utensils and, a supply of water for reconstitution, thus lowering their score. Nuts and dried fruits received 10 points as they require minimal dry storage space, have at least a 12 month shelf life, are not susceptible to bacterial contamination [10], do not require a heat source, cooking utensils or water for reconstitution, and have universal cultural acceptance. Dry milk in nitrogen-packed cans yielded seven points because it requires minimal dry storage space, has a 12 month shelf life and does not require a heat source; however, it requires utensils and a water supply for reconstitution [10].

**Table 3:** Scoring of food groups according to four main criteria for selecting foods for disaster response planning

Criteria	Dry cereals	Nuts	Dried fruits	Grains	Legumes	Dried meat/fish	Dry milk	Fresh vegetables	Fresh fruits	Fresh milk	Eggs	Fresh meat/fish
Nutrient Density	3	2	1	3	2	2	3	3	0	3	2	3
Storage/Handling properties	3	3	3	3	3	3	3	1	1	0	0	0
Low space requirement	√	√	√	√	√	√	√	-	-	-	-	-
Long shelf life (>12 months)	√	√	√	√	√	√	√	-	-	-	-	-
No refrigeration requirement/minimal sanitation concerns	√	√	√	√	√	√	√	√	√	-	-	-
Preparation ease	3	3	3	0	0	3	1	1	2	2	1	1
No utensils required	√	√	√	-	-	√	-	-	-	-	-	-
No cooking required	√	√	√	-	-	√	√	-	√	√	-	-
No reconstitution required	√	√	√	-	-	√	-	√	√	√	√	√
Cultural acceptance/individual tolerance	3	2	3	3	2	0	0	3	3	0	2	0
“Total” Score	12	10	10	9	7	8	7	8	6	5	5	4

### Disaster response diets (phase three)

The three DRDs developed were entitled *milk-inclusive, milk-free and Grab-and-Go* (Table 4). One to 3 servings of nuts (almonds, walnuts, cashews, pecans and pistachios) and dried fruits (apricots, figs, raisins, apples and dates) were included in all of the DRDs. The milk-inclusive DRD also featured 1 serving of dry milk, 5 servings of grains (white rice, cream of wheat, enriched pasta and saltine crackers) and 1 serving of legumes (raw peas, raw soybeans, canned kidney beans, cooked chickpeas and lentils). Lastly, the Grab-and-Go DRD included 3 servings of dry cereals (Corn Flakes®, Cheerios®, Shredded Wheat® and granola) in addition to the 3 servings of nuts and dried fruits. The nutritional goals that were being sought in developing the three DRDs were that they had to provide approximately 40–50 % of daily energy requirements and achieve a minimum of 40 % of the %DV for at least nine of the 14 nutrients from the NNR score.

**Table 4:** Composite of mean %Daily Values (%DV) for three proposed “Disaster Response Diets”

Nutrient	Milk-inclusive	Milk-free	Grab-and-Go
	5 Grains^a^	5 Grains^a^	3 Dry Cereals^b^
	1 Legumes^c^	1 Legumes^c^	3 Nuts^d^
	1 Nuts^d^	2 Nuts^d^	3 Dried Fruits^e^
	1 Dried Fruits^e^	1 Dried Fruits^e^	
	1 Dry Milk		
Energy	**43**	**47**	**40**
Protein	**50**	**44**	36
Vitamin A	29	18	37
Vitamin D	**325**	**300**	**600**
Vitamin E	18	30	**46**
Vitamin C	28	27	14
Vitamin B-1	**121**	**125**	**110**
Vitamin B-2	**104**	**80**	**93**
Folate	**105**	**106**	**99**
Vitamin B-12	**122**	**83**	**196**
Calcium	**44**	25	20
Iron	**111**	**116**	**88**
Zinc	**41**	**42**	**73**
Monounsaturated fatty acids	**42**	**78**	**113**
Potassium	38	32	**46**

Bold numbers are >40 % of the %DV

^a^Grains: cooked white rice (1/3 cup); 6 saltine crackers; cream of wheat, enriched pasta (1/2 cup cooked)

^b^Dry Cereals: Cornflakes®, Cheerios® (3/4 cup); Shredded Wheat® (1/2 cup); granola (1/4 cup)

^c^Legumes: raw peas, raw soybeans (1 cup); canned kidney beans, cooked chick peas, cooked lentils (1/2 cup)

^d^Nuts: almonds, walnuts, cashews, pecans, pistachios (1 ounce)

^e^Dried Fruits: apricots, figs, raisins, apples, dates (1/4 cup)

## Discussion

Nutrition professionals possess a unique set of skills that are imperative to the systems of coordination between disaster management departments and health departments in the provision of nutritional support in disasters. Our proposed three-phase multidimensional approach for creating a set of DRDs was designed to encompass important selection criteria of foods suitable for disasters; thereby improving upon then existing government recommendations for disaster response preparedness. Our approach builds upon nutrient density by considering food storage, preparation and cultural acceptance/individual tolerance.

There are no natural foods that contain all of the benchmark nutrients that are recommended for disaster response planning. However, an artificial formula-based food or ready to eat meals that meet a population’s nutritional requirements could be developed through food processing. Drawbacks to this approach are the cost burden and low acceptability of the artificial formula-based food or processed meals by the target population because most individuals prefer to consume “natural” foods [1]. On repeated occasions, artificial formula-based foods supplied during disasters have been only partially accepted or totally disregarded by the target population despite their nutritional needs. Specifically, food rations intended for human consumption have been fed to keep animals alive as a vital source of milk and cash [17]. Another consideration is that the target population may not enjoy the taste or texture of the artificial formula-based food or meals. This is important in that individuals may reject available food and put themselves at risk for nutritional deprivation. Therefore, we sought to create a set of DRDs based upon four important food selection criteria.

After evaluating the nutrient density of 11 specific food groups in Phase One, our scoring system during Phase Two found that five plant-based food groups (dry cereals, nuts, dried fruits, grains and legumes) achieved scores above midpoint because they possess favorable qualities in the context of our four distinct DRD planning criteria. Using plant-based foods has been recommended before by the Department of Defense in the manufacturing of Humanitarian Daily Rations for disaster relief [18].

A community that accepts dairy as part of their routine diet may consider choosing the milk-inclusive DRD because of the high Phase 2 score. If a population culturally accepts and tolerates dairy products, the “highest priority” food groups for a DRD are dry cereal, nuts, dried fruits, grains, legumes and dry milk, which will provide rich sources of energy, macronutrients and micronutrients. Furthermore, the simple addition of familiar spices/seasonings and condiments to plant-based foods may enhance cultural acceptance across societies. This is because spices do not require frequent rotation, are easy to store and are affordable.

An additional guideline for the amount of plant-based foods stored for disaster response preparedness is the prevalence of overweight and obesity within individual households, institutions, government agencies and NGOs. In light of the global prevalence of overweight and obesity, the set of DRDs that were developed have at least 11 less servings from the grain, fruit, vegetable, meat, milk and fat (e.g., nuts) groups that would be recommended to achieve 100 % or more of the Daily Value for energy and nutrients under “normal” times in the US. Therefore, the number of servings could be extrapolated by computing the daily energy needs of an individual household, institution, government agency or NGO based upon their current health statistics. Further, in light of the decreased energy needs in overweight and/or sedentary dwellers in an urban society, the daily energy requirement may be further evaluated to preserve scarce food resources for individuals performing rescue tasks and for those with greater energy needs, i.e. children and pregnant women.

A limitation of this current study is the use of the NNR score for evaluating nutrient density. The NNR score does not factor in the biological quality of the nutrients in the food source, their bioavailability, and the nutrient distribution in the food supply [12]. Additionally, the nutrients within the NNR score are not weighted based upon the aforementioned factors.

## Conclusions

In summary, a plant-based DRD can meet the nutritional requirements of adults and children residing in urban societies in the event of a disaster. Therefore, we suggest nutrition professionals consider using a plant-based approach for DRD planning for homes, institutions, government agencies and NGOs. Nuts and dried fruit meet the highest criteria for inclusion in designing DRDs for several reasons: they are energy dense, contribute outstanding nutritional value, are accepted by all cultures, are not proscribed by any religion, have a long shelf life, occupy minimal space, are easy to handle, require no manipulation or preparation before consumption, and are affordable. These factors make plant-based DRDs a practical and reliable form of nutrition during highly vulnerable circumstances.

## Abbreviations

DRD: Disaster response diet
DRI: Dietary reference intake
DV: Daily value
FEMA: Federal Emergency Management Agency
LF: Low fat
MUFA: Monounsaturated fatty acid
NF: Nonfat
NGO: Non-governmental organization
NNR: Naturally nutrient rich
US: United States
